# Supplementary material for: EmbryoNet: using deep learning to link embryonic phenotypes to signaling pathways
Source: Nat Methods. 2023 May 8;20(6):815–23. doi: 10.1038/s41592-023-01873-4 (PMC10250202; doi:10.1038/s41592-023-01873-4)
Supplement: Supplementary file 1 — Supplementary Notes 1–4 and References. [file 41592_2023_1873_MOESM1_ESM.pdf]

nature methods

Resource https://doi.org/10.1038/s41592-023-01873-4

EmbryoNet: using deep learning to link embryonic phenotypes to signaling pathways

In the format provided by the authors and unedited

Supplementary Note 1

Overview of developmental signaling pathways and early zebrafish embryogenesis

The zebrafish embryo develops from a single cell to a segmented larva with well-distinguishable organ primordia within 24 hpf[1] and is therefore a prime model system for our approach. After the cleavage stages, the future germ layers and the body axes become patterned during the blastula period. During gastrulation, these pre-determined cell populations migrate in a well-coordinated fashion to give rise to the later body-plan: The ectoderm and enveloping cell layer engulf the whole yolk sphere during epiboly, the presumptive mesoderm and endoderm ingress below the ectoderm to later form interior organs, and all tissues together move towards the dorsal side to establish the elongated shape of the embryo in a process called convergence and extension. In the following segmentation stages, the embryo elongates further, and somites and primary organs form[1].

The ligands of signaling pathways regulating these processes are dynamically expressed from specific source tissues in the embryo (Fig. 1a). Nodal expression starts in nuclei of the yolk-syncytial layer after the mid-blastula transition around 3 hpf and expands through the marginal blastoderm by positive feedback with peak expression around sphere stage at about 4 hpf[2,3]. FGF expression is induced by Nodal signaling and thus similarly localized around the margin starting from sphere stage[4,5]. During gastrulation, a second FGF expression domain in the future hindbrain is established[4]. BMP expression starts as a shallow ventral-to-dorsal gradient at late blastula stages around 4 hpf and steepens during gastrulation[6-9]. Canonical Wnt expression becomes visible around 5 hpf at 50% epiboly along the blastoderm margin[10]. Starting at mid-gastrulation, Wnt ligands are also expressed in the prospective neuro-epithelium, and the posterior expression domain is maintained in the dorsal paraxial tailbud region[10,11]. PCP ligands regulate convergence and extension movements and are expressed in the germ ring at shield stage around 6 hpf and in the paraxial mesoderm during gastrulation[12,13]. Sonic hedgehog is expressed first in the shield at 60% epiboly around 7 hpf, and expression continues in the notochord and the neural tube floorplate[14]. Retinoic acid is synthesized by the enzyme Raldh2, which is first detectable around 30% epiboly at about 5 hpf in the marginal zone and later in the posterior presomitic, somatic and lateral plate mesoderm[15].

Together, the expression patterns and activities of these signaling molecules cover the embryo (Fig. 1a) and jointly orchestrate the emergence of a body plan from an initially nearly uniform ball of cells.

Supplementary Note 2

Chemical genetics to modulate the activity of signaling pathways

Small molecules to modulate RA (e.g. Supplementary Ref.[16-18]), FGF (e.g. Supplementary Ref.[19-23]), Wnt (e.g. Supplementary Ref.[24-31]), Nodal (e.g. Supplementary Ref.[32-36]), BMP (e.g. Supplementary Ref.[37-42]) and Shh signaling (e.g. Supplementary Ref.[23,43-45]) have been validated for specificity and widely applied in previous studies. mRNA injections of pathway antagonists also induce *bona fide* signaling pathway loss-of-function phenotypes, and ectopically provided *lefty* and *chordin* mRNA can even rescue the respective zebrafish mutants (e.g. Supplementary Ref.[46-49]). In addition, the morpholino that we used to induce the - *PCP* phenotype has been extensively validated in previous studies (e.g. Supplementary Ref.[50-54]).

To further validate our approach, we directly compared phenotypes induced by small-molecule inhibitors, pathway antagonists or mutants. These were then classified by EmbryoNet. Nodal phenotypes induced by small-molecule inhibitor treatment (SB-505124, n = 33), injection of a pathway antagonist (*lefty1* mRNA, n = 27), or in a receptor mutant (MZ*oep*, n = 27) were all classified as - *Nodal* with similar accuracy (Extended Data Fig. 1f). Similarly, BMP phenotypes induced by small-molecule inhibitor treatment (LDN-193189, n = 45), pathway antagonist injection (*chordin* mRNA, n = 26), or in a pathway ligand mutant (*swirl*$^{-/-}$, n = 13), were all classified by EmbryoNet as - *BMP* with similar accuracy (Extended Data Fig. 1g). Importantly, - *BMP* phenotypes generated by overexpression of the BMP inhibitor Chordin were properly identified by EmbryoNet, even though such treatments had not been used for the training of the network. Furthermore, EmbryoNet recognized - *Shh* phenotypes with similar accuracy for both small-molecule treatment using Cyclopamine (82%) and *zGli3R-GFP* mRNA injection (72%).

Supplementary Note 3

Detection of known and novel developmental defect features by EmbryoNet-Prime

Using our class activation map (CAM) visualization approach (see Materials and Methods), we found that EmbryoNet-Prime often detected well-known features of defective signaling pathways. For example, it is well known that Wnt mutants have defective heads and tailbuds[11,55], and EmbryoNet-Prime was indeed positively activated in these regions at later stages, while during early segmentation the whole body axis showed positive activation (Supplementary Video 15). In embryos where the *dickkopf* enlarged head phenotype was less pronounced, the head displayed negative activation for - *Wnt* classification in agreement with human assessment (Supplementary Video 16). Interestingly, positive activation of the network in the head region was often restricted to the mid-hindbrain boundary, and positive activation in the tail region seemed to target the yolk extension and the space between tail and body, suggesting a potential role for the angle between the two structures. Most (7 of 10 analyzed) - *Wnt* embryos were detected earlier by EmbryoNet-Prime than by human assessors. In these cases, the CAM visualization approach showed positive activation in spots across and next to the embryo (Supplementary Video 16).

Similarly, early detection of - *Nodal* (5.5 hpf, Supplementary Video 19) was based on latent features not recognized by human assessors – primarily the border between yolk and blastoderm, and spots directly outside the embryo proper – while later classification was linked to established structures, such as the ectodermal thickening from late gastrulation, followed by positive activation in head, tail and trunk regions (Supplementary Video 20, $n = 10$). While the cyclopic eye showed positive activation in the - *Nodal* class, it interestingly remained neutral for - *Shh* embryos (Supplementary Videos 21-22, $n = 10$), where positive activation in the CAM visualization was apparent at the somites and yolk extension. Consequently, - *Shh* samples got classified at various times during somitogenesis.

- *BMP* embryos frequently (6 of 10 analyzed samples) first got detected in late gastrulation shortly before they start their characteristic elongation, accompanied by positive activation spots at the yolk (Fig. 3). When elongation started, the yolk, head and tail showed stronger positive activation, which was later maintained in head, tail or both (Supplementary Videos 11-12). Once - *BMP* embryos disintegrated, the classification immediately switched to negative activation.

Similarly, + *RA* was first identified at the end of gastrulation, when the area around the tailbud or the head was positively activated in CAM visualizations (Fig. 3, Supplementary Videos 13-14). Half of the analyzed samples ($n = 10$) were only classified once elongation became visible to some extent. Like in the - *BMP* class, tail and head stay frequently positive, with the signal often being situated directly outside the respective structure. Interestingly, the signal near the tail looked different from the one in - *Wnt* samples, supporting the idea that the angle between body and tail is sampled.

Most (7 of 10) *Normal* embryos were identified at the end of gastrulation, and these embryos displayed positive activation in the head and tailbud (Fig. 3, Supplementary Videos 9-10). A second wave of identification (3 of 10) occurred when somites became visible. In later development, the tail and the surrounding space were activated most consistently: In this case the movement of the embryo seemed to be identified.

The - *FGF* class was only reliably detected around 15 hpf, showing positive activation mostly in regions at the yolk and tail (Fig. 3, Supplementary Videos 17-18, $n = 10$). Interestingly, the tail later was neutral or even showed negative activation. Overall, negative activation was more dominant in - *FGF* embryos than in most other classes.

- *PCP* embryos often got classified correctly directly after gastrulation, when they showed mediolateral widening. The classification then tended to frequently change (Fig. 3, Supplementary Videos 23-24, $n = 10$) and only at late segmentation stages remained consistent. At these stages, the yolk extension and the somites displayed some positive activation. Overall, - *PCP* showed the least positive activation.

Supplementary Note 4

Use of statins in humans and in animal studies

For the last 40 years, statins have been the first-choice medication to treat hyperlipidemia in humans[56,57]. Statin treatment in pregnant women, however, is not recommended by most public health agencies due to potential teratogenic effects. For example, the Pharmacovigilance and Counseling Center for Embryonic Toxicology at the Charité supported by the German Federal Ministry of Health recommends to avoid the intake of atorvastatin and suggests the use of simvastatin if a therapy with statins cannot be circumvented (https://www.embryotox.de/arzneimittel/details/ansicht/medikament/atorvastatin, accessed on August 23rd 2022). However, in July 2021 the Food and Drug Administration (FDA) requested statin manufacturers to remove the FDA warning concerning statin usage during pregnancy (https://www.fda.gov/media/150774/download, accessed on August 23rd 2022).

A recent meta-analysis continues to suggest an association of statin use with premature delivery and decreased birth weight[58], whereas other reviews and meta-analyses have stated that there is no clear conclusion regarding the teratogenicity of statins[59-61]. Due to the lack of interventional studies in pregnant women, it is unclear whether teratogenic effects might depend on variables such as exposure duration or dosage. A potential effect of statins on FGF signaling, as observed in our zebrafish experiments (Fig. 4c-e), has previously been discussed for cultured human cells[62-66].

In previous animal studies, statins were applied during late organogenesis stages at doses much higher than those typically used in humans. The recommended daily human intake of atorvastatin ranges between 10 - 80 mg/d (0.13 - 1.0 mg/kg/d or 0.32 - 2.56 µM, assuming a body weight of 80 kg with 70% water content), for simvastatin between 5 - 80 mg/d (0.06 - 1.0 mg/kg/d or 0.21 - 3.41 µM, assuming a body weight of 80 kg with 70% water content) and for lovastatin between 20 - 60 mg/d (0.25 - 0.75 mg/kg/d or 0.88 - 2.65 µM, assuming a body weight of 80 kg with 70% water content). In contrast, doses in previous animal studies have typically used amounts between 2 and 200 mg/kg/d, ranging from micro- to millimolar daily concentrations. When applied during late organogenesis stages in the gestation period, no teratogenicity of atorvastatin could be found in rats, whereas a slight tendency for increased fetal loss and decreased birth weight was observed in rabbits[67]. Similar results were obtained for simvastatin and lovastatin[68]. However, data for statin exposure at earlier embryonic stages is not available.

In our zebrafish embryo experiments, strong dorsal-ventral patterning defects (Fig. 4c) were evident at concentrations comparable to human therapeutic doses and as low as 0.4 µM (see Materials and Methods). However, the bioavailability in zebrafish embryos compared to human cells and tissues is currently unclear.

Supplementary References

1 Kimmel, C. B., Ballard, W. W., Kimmel, S. R., Ullmann, B. & Schilling, T. F. Stages of embryonic development of the zebrafish. *Dev Dyn* **203**, 253-310 (1995). https://doi.org:10.1002/aja.1002030302

2 Rebagliati, M. R., Toyama, R., Fricke, C., Haffter, P. & Dawid, I. B. Zebrafish nodal-related genes are implicated in axial patterning and establishing left-right asymmetry. *Dev Biol* **199**, 261-272 (1998). https://doi.org:10.1006/dbio.1998.8935

3 Feldman, B. *et al.* Zebrafish organizer development and germ-layer formation require nodal-related signals. *Nature* **395**, 181-185 (1998). https://doi.org:10.1038/26013

4 Fürthauer, M., Thisse, C. & Thisse, B. A role for FGF-8 in the dorsoventral patterning of the zebrafish gastrula. *Development* **124**, 4253-4264 (1997). https://doi.org:10.1242/dev.124.21.4253

5 Mathieu, J. *et al.* Nodal and Fgf pathways interact through a positive regulatory loop and synergize to maintain mesodermal cell populations. *Development* **131**, 629-641 (2004). https://doi.org:10.1242/dev.00964

6 Pomreinke, A. P. *et al.* Dynamics of BMP signaling and distribution during zebrafish dorsal-ventral patterning. *Elife* **6**, e25861 (2017). https://doi.org:10.7554/eLife.25861

7 Ramel, M. C. & Hill, C. S. The ventral to dorsal BMP activity gradient in the early zebrafish embryo is determined by graded expression of BMP ligands. *Dev Biol* **378**, 170-182 (2013). https://doi.org:10.1016/j.ydbio.2013.03.003

8 Tucker, J. A., Mintzer, K. A. & Mullins, M. C. The BMP signaling gradient patterns dorsoventral tissues in a temporally progressive manner along the anteroposterior axis. *Dev Cell* **14**, 108-119 (2008). https://doi.org:10.1016/j.devcel.2007.11.004

9 Zinski, J. *et al.* Systems biology derived source-sink mechanism of BMP gradient formation. *Elife* **6** (2017). https://doi.org:10.7554/eLife.22199

10 Kelly, G. M., Greenstein, P., Erezyilmaz, D. F. & Moon, R. T. Zebrafish wnt8 and wnt8b share a common activity but are involved in distinct developmental pathways. *Development* **121**, 1787-1799 (1995). https://doi.org:10.1242/dev.121.6.1787

11 Lekven, A. C., Thorpe, C. J., Waxman, J. S. & Moon, R. T. Zebrafish wnt8 encodes two wnt8 proteins on a bicistronic transcript and is required for mesoderm and neurectoderm patterning. *Dev Cell* **1**, 103-114 (2001). https://doi.org:10.1016/s1534-5807(01)00007-7

12 Heisenberg, C. P. *et al.* Silberblick/Wnt11 mediates convergent extension movements during zebrafish gastrulation. *Nature* **405**, 76-81 (2000). https://doi.org:10.1038/35011068

13 Kilian, B. *et al.* The role of Ppt/Wnt5 in regulating cell shape and movement during zebrafish gastrulation. *Mech Dev* **120**, 467-476 (2003). https://doi.org:10.1016/s0925-4773(03)00004-2

14 Krauss, S., Concordet, J. P. & Ingham, P. W. A functionally conserved homolog of the Drosophila segment polarity gene hh is expressed in tissues with polarizing activity in zebrafish embryos. *Cell* **75**, 1431-1444 (1993). https://doi.org:10.1016/0092-8674(93)90628-4

15 Begemann, G., Schilling, T. F., Rauch, G. J., Geisler, R. & Ingham, P. W. The zebrafish neckless mutation reveals a requirement for raldh2 in mesodermal signals that pattern

the hindbrain. *Development* **128**, 3081-3094 (2001). https://doi.org:10.1242/dev.128.16.3081

16 Dalgin, G. *et al.* Zebrafish mnx1 controls cell fate choice in the developing endocrine pancreas. *Development* **138**, 4597-4608 (2011). https://doi.org:10.1242/dev.067736

17 D'Aniello, E., Ravisankar, P. & Waxman, J. S. Rdh10a provides a conserved critical step in the synthesis of retinoic acid during zebrafish embryogenesis. *PLoS One* **10**, e0138588 (2015). https://doi.org:10.1371/journal.pone.0138588

18 Franzosa, J. A. *et al.* Retinoic acid-dependent regulation of miR-19 expression elicits vertebrate axis defects. *FASEB J* **27**, 4866-4876 (2013). https://doi.org:10.1096/fj.12-225524

19 Sun, L. *et al.* Design, synthesis, and evaluations of substituted 3-[(3- or 4-carboxyethylpyrrol-2-yl)methylidenyl]indolin-2-ones as inhibitors of VEGF, FGF, and PDGF receptor tyrosine kinases. *J Med Chem* **42**, 5120-5130 (1999). https://doi.org:10.1021/jm9904295

20 Rohner, N. *et al.* Duplication of fgfr1 permits Fgf signaling to serve as a target for selection during domestication. *Curr Biol* **19**, 1642-1647 (2009). https://doi.org:10.1016/j.cub.2009.07.065

21 Maier, E. C. & Whitfield, T. T. RA and FGF signalling are required in the zebrafish otic vesicle to pattern and maintain ventral otic identities. *PLoS Genet* **10**, e1004858 (2014). https://doi.org:10.1371/journal.pgen.1004858

22 Lovely, C. B., Swartz, M. E., McCarthy, N., Norrie, J. L. & Eberhart, J. K. Bmp signaling mediates endoderm pouch morphogenesis by regulating Fgf signaling in zebrafish. *Development* **143**, 2000-2011 (2016). https://doi.org:10.1242/dev.129379

23 Osborn, D. P. S. *et al.* Fgf-driven Tbx protein activities directly induce myf5 and myod to initiate zebrafish myogenesis. *Development* **147** (2020). https://doi.org:10.1242/dev.184689

24 Wang, X. *et al.* The development of highly potent inhibitors for porcupine. *J Med Chem* **56**, 2700-2704 (2013). https://doi.org:10.1021/jm400159c

25 Grainger, S. *et al.* Wnt9a is required for the aortic amplification of nascent hematopoietic stem cells. *Cell Rep* **17**, 1595-1606 (2016). https://doi.org:10.1016/j.celrep.2016.10.027

26 Takayama, K., Muto, A. & Kikuchi, Y. Leucine/glutamine and v-ATPase/lysosomal acidification via mTORC1 activation are required for position-dependent regeneration. *Sci Rep* **8**, 8278 (2018). https://doi.org:10.1038/s41598-018-26664-2

27 Kamei, C. N., Gallegos, T. F., Liu, Y., Hukriede, N. & Drummond, I. A. Wnt signaling mediates new nephron formation during zebrafish kidney regeneration. *Development* **146** (2019). https://doi.org:10.1242/dev.168294

28 Nie, C. H. *et al.* Development of teleost intermuscular bones undergoing intramembranous ossification based on histological-transcriptomic-proteomic data. *Int J Mol Sci* **20** (2019). https://doi.org:10.3390/ijms20194698

29 Narumi, R., Liu, S., Ikeda, N., Morita, O. & Tasaki, J. Chemical-induced cleft palate is caused and rescued by pharmacological modulation of the canonical Wnt signaling pathway in a zebrafish model. *Front Cell Dev Biol* **8**, 592967 (2020). https://doi.org:10.3389/fcell.2020.592967

30 Kalantary-Charvadeh, A., Hosseini, V., Mehdizadeh, A. & Darabi, M. Application of porcupine inhibitors in stem cell fate determination. *Chem Biol Drug Des* **96**, 1052-1068 (2020). https://doi.org:10.1111/cbdd.13704

31 Nile, A. H. & Hannoush, R. N. Fatty acylation of Wnt proteins. *Nat Chem Biol* **12**, 60-69 (2016). https://doi.org:10.1038/nchembio.2005

32 DaCosta Byfield, S., Major, C., Laping, N. J. & Roberts, A. B. SB-505124 is a selective inhibitor of transforming growth factor-beta type I receptors ALK4, ALK5, and ALK7. *Mol Pharmacol* **65**, 744-752 (2004). https://doi.org:10.1124/mol.65.3.744

33 Deshwar, A. R., Chng, S. C., Ho, L., Reversade, B. & Scott, I. C. The Apelin receptor enhances Nodal/TGFbeta signaling to ensure proper cardiac development. *Elife* **5** (2016). https://doi.org:10.7554/eLife.13758

34 Terashima, A. V., Mudumana, S. P. & Drummond, I. A. Odd skipped related 1 is a negative feedback regulator of nodal-induced endoderm development. *Dev Dyn* **243**, 1571-1580 (2014). https://doi.org:10.1002/dvdy.24191

35 Gonsar, N. *et al.* Temporal and spatial requirements for Nodal-induced anterior mesendoderm and mesoderm in anterior neurulation. *Genesis* **54**, 3-18 (2016). https://doi.org:10.1002/dvg.22908

36 van Boxtel, A. L., Economou, A. D., Heliot, C. & Hill, C. S. Long-range signaling activation and local inhibition separate the mesoderm and endoderm lineages. *Dev Cell* **44**, 179-191 e175 (2018). https://doi.org:10.1016/j.devcel.2017.11.021

37 Cuny, G. D. *et al.* Structure-activity relationship study of bone morphogenetic protein (BMP) signaling inhibitors. *Bioorg Med Chem Lett* **18**, 4388-4392 (2008). https://doi.org:10.1016/j.bmcl.2008.06.052

38 Steinbicker, A. U. *et al.* Inhibition of bone morphogenetic protein signaling attenuates anemia associated with inflammation. *Blood* **117**, 4915-4923 (2011). https://doi.org:10.1182/blood-2010-10-313064

39 Cannon, J. E., Upton, P. D., Smith, J. C. & Morrell, N. W. Intersegmental vessel formation in zebrafish: requirement for VEGF but not BMP signalling revealed by selective and non-selective BMP antagonists. *Br J Pharmacol* **161**, 140-149 (2010). https://doi.org:10.1111/j.1476-5381.2010.00871.x

40 Zinck, N. W., Jeradi, S. & Franz-Odendaal, T. A. Elucidating the early signaling cues involved in zebrafish chondrogenesis and cartilage morphology. *J Exp Zool B Mol Dev Evol* **336**, 18-31 (2021). https://doi.org:10.1002/jez.b.23012

41 Kruse-Bend, R. *et al.* Extraocular ectoderm triggers dorsal retinal fate during optic vesicle evagination in zebrafish. *Dev Biol* **371**, 57-65 (2012). https://doi.org:10.1016/j.ydbio.2012.08.004

42 Place, E. S. & Smith, J. C. Zebrafish atoh8 mutants do not recapitulate morpholino phenotypes. *PLoS One* **12**, e0171143 (2017). https://doi.org:10.1371/journal.pone.0171143

43 Incardona, J. P., Gaffield, W., Kapur, R. P. & Roelink, H. The teratogenic Veratrum alkaloid cyclopamine inhibits sonic hedgehog signal transduction. *Development* **125**, 3553-3562 (1998). https://doi.org:10.1242/dev.125.18.3553

44 Quint, E. *et al.* Bone patterning is altered in the regenerating zebrafish caudal fin after ectopic expression of sonic hedgehog and bmp2b or exposure to cyclopamine. *Proc Natl Acad Sci U S A* **99**, 8713-8718 (2002). https://doi.org:10.1073/pnas.122571799

45 Muthu, V., Eachus, H., Ellis, P., Brown, S. & Placzek, M. Rx3 and Shh direct anisotropic growth and specification in the zebrafish tuberal/anterior hypothalamus. *Development* **143**, 2651-2663 (2016). https://doi.org:10.1242/dev.138305

46 Fisher, S. & Halpern, M. E. Patterning the zebrafish axial skeleton requires early chordin function. *Nat Genet* **23**, 442-446 (1999). https://doi.org:10.1038/70557

47 Rogers, K. W. *et al.* Nodal patterning without Lefty inhibitory feedback is functional but fragile. *Elife* **6** (2017). https://doi.org:10.7554/eLife.28785

48 Schulte-Merker, S., Lee, K. J., McMahon, A. P. & Hammerschmidt, M. The zebrafish organizer requires chordino. *Nature* **387**, 862-863 (1997). https://doi.org:10.1038/43092

49 Tuazon, F. B., Wang, X., Andrade, J. L., Umulis, D. & Mullins, M. C. Proteolytic restriction of chordin range underlies BMP gradient formation. *Cell Rep* **32**, 108039 (2020). https://doi.org:10.1016/j.celrep.2020.108039

50 Williams, B. B. *et al.* VANGL2 regulates membrane trafficking of MMP14 to control cell polarity and migration. *J Cell Sci* **125**, 2141-2147 (2012). https://doi.org:10.1242/jcs.097964

51 Williams, M. L. & Solnica-Krezel, L. Nodal and planar cell polarity signaling cooperate to regulate zebrafish convergence and extension gastrulation movements. *Elife* **9** (2020). https://doi.org:10.7554/eLife.54445

52 Prince, D. J. & Jessen, J. R. Dorsal convergence of gastrula cells requires Vangl2 and an adhesion protein-dependent change in protrusive activity. *Development* **146** (2019). https://doi.org:10.1242/dev.182188

53 Love, A. M., Prince, D. J. & Jessen, J. R. Vangl2-dependent regulation of membrane protrusions and directed migration requires a fibronectin extracellular matrix. *Development* **145** (2018). https://doi.org:10.1242/dev.165472

54 Johansson, M., Giger, F. A., Fielding, T. & Houart, C. Dkk1 controls cell-cell interaction through regulation of non-nuclear beta-catenin pools. *Dev Cell* **51**, 775-786 e773 (2019). https://doi.org:10.1016/j.devcel.2019.10.026

55 Hino, H. *et al.* Roles of maternal wnt8a transcripts in axis formation in zebrafish. *Dev Biol* **434**, 96-107 (2018). https://doi.org:10.1016/j.ydbio.2017.11.016

56 Ruscica, M., Ferri, N., Banach, M., Sirtori, C. R. & Corsini, A. Side effects of statins-from pathophysiology and epidemiology to diagnostic and therapeutic implications. *Cardiovasc Res* (2022). https://doi.org:10.1093/cvr/cvac020

57 Zhao, G. *et al.* Effect of statins use on risk and prognosis of breast cancer: a meta-analysis. *Anticancer Drugs* **33**, e507-e518 (2022). https://doi.org:10.1097/CAD.0000000000001151

58 Karadas, B. *et al.* Pregnancy outcomes following maternal exposure to statins: A systematic review and meta-analysis. *Br J Clin Pharmacol* **88**, 3962-3976 (2022). https://doi.org:10.1111/bcp.15423

59 Vahedian-Azimi, A., Makvandi, S., Banach, M., Reiner, Z. & Sahebkar, A. Fetal toxicity associated with statins: A systematic review and meta-analysis. *Atherosclerosis* **327**, 59-67 (2021). https://doi.org:10.1016/j.atherosclerosis.2021.05.006

60 Kusters, D. M. *et al.* Statin use during pregnancy: a systematic review and meta-analysis. *Expert Rev Cardiovasc Ther* **10**, 363-378 (2012). https://doi.org:10.1586/erc.11.196

61 Zarek, J. & Koren, G. The fetal safety of statins: a systematic review and meta-analysis. *J Obstet Gynaecol Can* **36**, 506-509 (2014). https://doi.org:10.1016/S1701-2163(15)30565-X

62 Burgazli, K. M. *et al.* The impact of statins on FGF-2-stimulated human umbilical vein endothelial cells. *Postgrad Med* **126**, 118-128 (2014). https://doi.org:10.3810/pgm.2014.01.2732

63 Shiota, M. *et al.* Pravastatin-induced proangiogenic effects depend upon extracellular FGF-2. *J Cell Mol Med* **16**, 2001-2009 (2012). https://doi.org:10.1111/j.1582-4934.2011.01494.x

64 Fafilek, B. *et al.* Statins do not inhibit the FGFR signaling in chondrocytes. *Osteoarthritis Cartilage* **25**, 1522-1530 (2017). https://doi.org:10.1016/j.joca.2017.05.014

65 Yamashita, A. *et al.* Statin treatment rescues FGFR3 skeletal dysplasia phenotypes. *Nature* **513**, 507-511 (2014). https://doi.org:10.1038/nature13775

66 Park, H. J. *et al.* Human umbilical vein endothelial cells and human dermal microvascular endothelial cells offer new insights into the relationship between lipid metabolism and angiogenesis. *Stem Cell Rev* **2**, 93-102 (2006). https://doi.org:10.1007/s12015-006-0015-x

67 Dostal, L. A., Schardein, J. L. & Anderson, J. A. Developmental toxicity of the HMG-CoA reductase inhibitor, atorvastatin, in rats and rabbits. *Teratology* **50**, 387-394 (1994). https://doi.org:10.1002/tera.1420500604

68 Manson, J. M., Freyssinges, C., Ducrocq, M. B. & Stephenson, W. P. Postmarketing surveillance of lovastatin and simvastatin exposure during pregnancy. *Reprod Toxicol* **10**, 439-446 (1996). https://doi.org:10.1016/s0890-6238(96)00130-x
