# Supplementary material for: EmbryoNet: using deep learning to link embryonic phenotypes to signaling pathways
Source: Nat Methods. 2023 May 8;20(6):815–23. doi: 10.1038/s41592-023-01873-4 (PMC10250202; doi:10.1038/s41592-023-01873-4)
Supplement: Supplementary file 2 — Reporting Summary [file 41592_2023_1873_MOESM2_ESM.pdf]

nature portfolio

Corresponding author(s): Patrick Müller

Last updated by author(s): Mar 22, 2023

Reporting Summary

Nature Portfolio wishes to improve the reproducibility of the work that we publish. This form provides structure for consistency and transparency in reporting. For further information on Nature Portfolio policies, see our Editorial Policies and the Editorial Policy Checklist.

Statistics

For all statistical analyses, confirm that the following items are present in the figure legend, table legend, main text, or Methods section.

n/a	Confirmed	
☐	☒	The exact sample size (*n*) for each experimental group/condition, given as a discrete number and unit of measurement
☐	☒	A statement on whether measurements were taken from distinct samples or whether the same sample was measured repeatedly
☒	☐	The statistical test(s) used AND whether they are one- or two-sided<br>*Only common tests should be described solely by name; describe more complex techniques in the Methods section.*
☒	☐	A description of all covariates tested
☒	☐	A description of any assumptions or corrections, such as tests of normality and adjustment for multiple comparisons
☐	☒	A full description of the statistical parameters including central tendency (e.g. means) or other basic estimates (e.g. regression coefficient) AND variation (e.g. standard deviation) or associated estimates of uncertainty (e.g. confidence intervals)
☒	☐	For null hypothesis testing, the test statistic (e.g. *F*, *t*, *r*) with confidence intervals, effect sizes, degrees of freedom and *P* value noted<br>*Give P values as exact values whenever suitable.*
☒	☐	For Bayesian analysis, information on the choice of priors and Markov chain Monte Carlo settings
☒	☐	For hierarchical and complex designs, identification of the appropriate level for tests and full reporting of outcomes
☒	☐	Estimates of effect sizes (e.g. Cohen's *d*, Pearson's *r*), indicating how they were calculated

Our web collection on statistics for biologists contains articles on many of the points above.

Software and code

Policy information about availability of computer code

Data collection	For data acquisition on an Acquifer Imaging Machine, we used the Imaging Machine control software (Acquifer, Version ID 4.00.21). For data collection on a Keyence BZ-X810 microscope, we used the BZ-X800 viewer (Keyence, Version 01.03.00.01). For data acquisition on a ZEISS Lightsheet Z.1 microscope, we used ZEN 3.1 Black Edition (ZEISS).
Data analysis	For image annotation, model training, validation and testing we used our custom software EmbryoNet (http://github.com/mueller-lab/EmbryoNet, http://doi.org/10.5281/zenodo.7531593). For the analysis of pErk stainings, we used Fiji/ImageJ version 1.53 and MATLAB (R2022a). The training was performed on an NVIDIA RTX 3090 card in Ubuntu 20.04.4 LTS.

For manuscripts utilizing custom algorithms or software that are central to the research but not yet described in published literature, software must be made available to editors and reviewers. We strongly encourage code deposition in a community repository (e.g. GitHub). See the Nature Portfolio guidelines for submitting code & software for further information.

Data

Policy information about availability of data

All manuscripts must include a data availability statement. This statement should provide the following information, where applicable:

- Accession codes, unique identifiers, or web links for publicly available datasets
- A description of any restrictions on data availability
- For clinical datasets or third party data, please ensure that the statement adheres to our policy

Training and evaluation data sets for EmbryoNet are available from http://embryonet.uni-konstanz.de and http://doi.org/10.48606/15. The drug screen data is available from http://doi.org/10.48606/37, http://doi.org/10.48606/38 and http://doi.org/10.48606/41. Additional data that support the findings of this study are available from http://doi.org/10.48606/53 and http://doi.org/10.48606/55.

Human research participants

Policy information about studies involving human research participants and Sex and Gender in Research.

Reporting on sex and gender	N/A
Population characteristics	N/A
Recruitment	N/A
Ethics oversight	N/A

Note that full information on the approval of the study protocol must also be provided in the manuscript.

Field-specific reporting

Please select the one below that is the best fit for your research. If you are not sure, read the appropriate sections before making your selection.

☒ Life sciences ☐ Behavioural & social sciences ☐ Ecological, evolutionary & environmental sciences

For a reference copy of the document with all sections, see nature.com/documents/nr-reporting-summary-flat.pdf

Life sciences study design

All studies must disclose on these points even when the disclosure is negative.

Sample size	To determine a suitable sample size for the development of EmbryoNet, we used an active learning approach. In an iterative process, we progressively increased the number of images/embryos used as training and validation sets until the classification performance on the validation data set reached a saturation level. This approach is commonly used in classification problems and allows to obtain good classification performance. For all other experiments, at least three biological replicates were estimated to provide an adequate sample size based on previous analyses (Pomreinke et al. eLife 2017, Soh et al. Cell Reports 2020, Kuhn et al. Nature Communications 2022).
Data exclusions	Embryos that were only partially visible in images were excluded. For the analysis of pERK, only images of embryos that were oriented with the dorsal side facing the camera were used. The dorsal side could be identified after generating maximum intensity projections from image stacks. Embryos with tilted dorso-ventral axes were excluded.
Replication	The classification experiment with mixed MZoep and WT embryos comprises four biological replicates performed on the same day with multiple embryos. The heterozygous swirl mutant incross experiment has six biological replicates performed on the same day with multiple embryos. The lft1 and chordin overexpression experiments consist of nine biological replicates performed on the same day with multiple embryos. The classification of a stack of 98 selected embryos was performed once for random classification, 55 times by non-expert teams (31 without time information, 24 with extra time information), and by one experienced developmental biologist. The classification of time-lapse data sets was performed by two experienced developmental biologists once each. All automatic classifications are from one repetition by EmbryoNet or by EmbryoNet-Prime, respectively. The drug screen was performed once with multiple embryos for each treatment. The Statin findings were confirmed twice independently on separate days. The pERK immunostainings were performed with multiple embryos on the same day.
Randomization	Embryos from zebrafish and medaka crosses as well as stickleback in vitro-fertilizations were randomly allocated into experimental groups.
Blinding	Since embryos from zebrafish and medaka crosses as well as stickleback in-vitro fertilizations were genetically uniform and indistinguishable, blinding of the investigators was not necessary.

Reporting for specific materials, systems and methods

We require information from authors about some types of materials, experimental systems and methods used in many studies. Here, indicate whether each material, system or method listed is relevant to your study. If you are not sure if a list item applies to your research, read the appropriate section before selecting a response.

Materials & experimental systems

n/a	Involved in the study
☐	☒ Antibodies
☒	☐ Eukaryotic cell lines
☒	☐ Palaeontology and archaeology
☐	☒ Animals and other organisms
☒	☐ Clinical data
☒	☐ Dual use research of concern

Methods

n/a	Involved in the study
☒	☐ ChIP-seq
☒	☐ Flow cytometry
☒	☐ MRI-based neuroimaging

Antibodies

Antibodies used	We used anti-DP-ERK (Sigma-Aldrich, M8159) antibody at a dilution of 1:5000, and HRP-conjugated anti-mouse (Jackson ImmunoResearch, 715-035-150) antibody at a dilution of 1:5000.
Validation	We used a validated primary antibody from a standard commercial source (for validation see for example Rogers et al. eLife 2020, Navon et al. J Mol Neurosci 2012, Larbuisson et al. Differentiation 2013). The secondary antibody was also from a standard commercial source (for validation see e.g. Kim et al. Nature Communications 2022, Rogers et al. eLife 2020, Pardi et al. Nature Communications 2022).

Animals and other research organisms

Policy information about studies involving animals; ARRIVE guidelines recommended for reporting animal research, and Sex and Gender in Research

Laboratory animals	The experiments were performed exclusively with embryos and larvae that were not yet freely feeding. We used wild-type zebrafish, medaka and stickleback embryos. In addition, swirl mutants (Kishimoto et al. 1997) and maternal-zygotic oep zebrafish mutants were used (Gritsman et al. Cell 1999). Age of embryos: zebrafish (TE, oep and swr strains): 0-48 hpf; medaka (Cab strain): 0-48 hpf; stickleback (Little Campbell River and Tyne River strains): 0-140 hpf.
Wild animals	We did not use wild animals.
Reporting on sex	Sex-based analysis was not performed because phenotypical sex identification is not possible in zebrafish, medaka or stickleback embryos.
Field-collected samples	We did not use field-collected samples.
Ethics oversight	All procedures were executed in accordance with the guidelines of the EU directive 2010/63/EU and the German Animal Welfare Act as approved by the local authorities represented by the Regierungspräsidium Tübingen and the Regierungspräsidium Freiburg (Baden-Württemberg, Germany).

Note that full information on the approval of the study protocol must also be provided in the manuscript.
